# Spontaneous playful teasing in four great ape species

**DOI:** 10.1098/rspb.2023.2345

**Published:** 2024-02-14

**Authors:** I. B. Laumer, S. L. Winkler, F. Rossano, E. A. Cartmill

**Affiliations:** ^1^ Department of Anthropology, University of California Los Angeles, Haines Hall, Los Angeles, CA 90095, USA; ^2^ Max Planck Institute of Animal Behavior, Development and Evolution of Cognition, Konstanz, Germany; ^3^ Department of Cognitive Science, University of California San Diego, San Diego, USA; ^4^ Department of Anthropology, Cognitive Science Program, Indiana University, Bloomington, USA

**Keywords:** playful teasing, humour, social cognition, play, non-human primate, great ape

## Abstract

Joking draws on complex cognitive abilities: understanding social norms, theory of mind, anticipating others' responses and appreciating the violation of others’ expectations. Playful teasing, which is present in preverbal infants, shares many of these cognitive features. There is some evidence that great apes can tease in structurally similar ways, but no systematic study exists. We developed a coding system to identify playful teasing and applied it to video of zoo-housed great apes. All four species engaged in intentionally provocative behaviour, frequently accompanied by characteristics of play. We found playful teasing to be characterized by attention-getting, one-sidedness, response looking, repetition and elaboration/escalation. It takes place mainly in relaxed contexts, has a wide variety of forms, and differs from play in several ways (e.g. asymmetry, low rates of play signals like the playface and absence of movement-final ‘holds’ characteristic of intentional gestures). As playful teasing is present in all extant great ape genera, it is likely that the cognitive prerequisites for joking evolved in the hominoid lineage at least 13 million years ago.

## 1. Background

Joking is an important part of human interaction (e.g. [1]), one that draws on social intelligence, an ability to anticipate future actions and an ability to recognize and appreciate the violation of others' expectations. Teasing has much in common with joking, and *playful teasing* may be seen as a cognitive precursor. Like joking, playful teasing walks a fine line between aggression and play; it is mutually enjoyable, occurs in close relationships, requires the anticipation of another's response and involves creating ‘unexpected’ moments that deviate from expected interaction norms [2]. Playful teasing has the potential to create mutual amusement, which may serve to maintain or strengthen social bonds [3,4]. Since teasing is highly ambiguous and includes elements of both play and aggression, the teaser needs to anticipate how the recipient is likely to perceive the teasing act. For a teasing act to be interpreted as playful rather than aggressive, the recipient must be able to correctly identify the teaser's benign or playful intent [5], particularly when the actions used to tease are also used in aggressive contexts. The interpretation of actions during playful teasing (and play more broadly) as ‘not-serious’ may be one of the simplest and earliest forms of pretense [6].

Playful teasing emerges very early in human childhood. Infants playfully tease others even before they produce their first words, starting as young as 8 months old [7,8]. While the playful teasing of human infants is certainly not as cognitively complex as adult forms of joking, it seems likely that the social and conceptual building blocks of joking (understanding others' expectations and deriving enjoyment from violating them) are already present in infants’ teasing. Vasu Reddy and colleagues found that by 12 months, human infants produce three types of playful teasing behaviours [2,6–10]. These are *offer and withdrawal of objects* (offering an object and quickly pulling it back), *provocative non-compliance* (attempting to perform a prohibited action or refusing to perform an expected behaviour) and *disrupting others' activities* (e.g. blocking another's path or taking objects when others attempt to use them; also see [11,12]). Reddy reported that infants usually repeated these behaviours several times, while looking towards their parent, smiling and waiting for an emotional response. Importantly, infants seemed to seek positive responses; actions that led to negative emotional responses were rarely repeated [8]. Infants typically teased others in moments of neutrality or boredom, seemingly to initiate and maintain playful interactions while also exploring social limits [2,12,13].

Since playful teasing is observed in preverbal human infants, language cannot be a prerequisite. This opens up the possibility that similar forms of playful teasing might be present in non-human animals. Apes are excellent candidates for playful teasing, as all ape species laugh [14,15], engage in social object play [16], and display relatively sophisticated understandings of others' expectations [17–19]. Using a survey of the primate literature and data from our past research, we previously reported that great apes engage in the same three forms of playful teasing seen in preverbal human infants [5]. The current study widens the lens beyond these forms and analyses new video of ape interactions to ask how apes tease one another in playful ways.

Teasing behaviour in great apes and other animals is drastically understudied [5]. So far, ape teasing has only been systematically studied in zoo-living chimpanzees [20–22], and has focused only on agonistic forms of teasing (labelled ‘harassment’). Agonistic teasing has been described as a means for chimpanzees to learn about social rules or norms and to improve or reinforce position in the hierarchy [20–23]. These studies on agonistic teasing explicitly excluded behaviours accompanied by play signals (e.g. playface). By contrast, studies of play in great apes usually focus on the biological function and development of play rather than the interactional dynamics (e.g. [24]). The relative dearth of playful teasing descriptions in the animal literature (see [23,25,26]) seems to result from a systematic bias to exclude provocative interactions associated with play [5]. This could be due either to a desire to focus only on clearly affiliative or agonistic behaviours, or to methodological difficulties in measuring play.

Disentangling playful teasing from ‘pure’ play or aggression is challenging, as teasing is a highly ambiguous behaviour that straddles the border between play and aggression and can display behavioural characteristics of both [5]. In play, signals such as the playface are thought to inhibit aggression and coordinate playful behaviour [27–29]. Play signals during teasing might serve to reduce uncertainty in the recipient and signal a playful mood. In human infants, the presence of play markers has been used to argue that playful teasing might serve a bond-strengthening function [8]. Our study is not able to assess the function of playful teasing in apes, but by developing a coding system to define and study playful teasing from a bottom-up perspective, we hope to enable future analyses of the function of this highly ambiguous behaviour.

Using ape gesture coding systems as a starting point, we developed an extensive behavioural coding system aimed at identifying, describing and quantifying playful teasing behaviours in great apes. As in studies of gesture, we tried to identify the actors' and recipients’ actions, goal-directedness, bodily movements and facial expressions, and the recipients' behavioural responses to actions [30–33]. We also coded markers of intentionality: directedness, response waiting, persistence and elaboration [31,32,34]. To identify potential teasing events, we focused on videos of spontaneous non-aggressive social interactions. Our goal was to design a coding system and criteria for identifying playful teasing in order to distinguish this behaviour from the explicitly agonistic interactions reported in previous literature. We hope that this work results in a better understanding of the forms, functions and outcomes of affiliative, provocative behaviours in apes.

## 2. Methods

### (a) Subjects and housing

The video corpus was recorded during observations of groups of nine bonobos (*Pan paniscus*), four orangutans (*Pongo abelii*) and four gorillas (*Gorilla gorilla* and *Gorilla beringei*) at San Diego Zoo, and a group of 17 chimpanzees (*Pan troglodytes*) at Leipzig Zoo. Apes were housed in enriched, indoor-outdoor enclosures and were fed several times a day. For detailed subject information and housing see electronic supplementary material, section A.

### (b) Description of the video corpus

We used a video corpus of four groups of zoo-housed great apes: chimpanzees, bonobos, gorillas, and orangutans. The videos were collected by FR and colleagues from 2016 to 2019, and consisted of focal follows of four juveniles, three to five years of age, one from each species group. For detailed information about group composition and housing, please see electronic supplementary material, section A, table S1. Each group had only one juvenile, except the chimpanzees, which had two. Although the videos followed the four focal individuals, other group members were typically visible. Animal identification was performed by comparing videos to pictures of all group individuals; in the few cases where an individual could not be identified, age and sex class were recorded.

We coded all visible social interactions on the videos, not just those involving the focal subjects. However, the focal juvenile was involved in the vast majority of the teasing events in this corpus. The focal juvenile was involved in 80% of the chimpanzee teasing events and 100% of the bonobo, gorilla, and orangutan teasing events. The focal bonobo was the teaser 85% of the time and the target 15% of the time. For gorillas and orangutans, the focal juvenile was the teaser in all teasing events. Several of the chimpanzee teasing events involved the non-focal juvenile Ohini interacting with adults. In our final dataset, Ohini was the teaser 11% of the time and the target 7% of the time.

Although the videos were gathered using focal sampling, our construction of a teasing video corpus was more akin to ad libitum sampling, since we also coded the behaviours of non-focal individuals and thus could not calculate rates of teasing. Similar to Adang [20–22], the goal of this project was to identify a type of behaviour and build an initial data set. While we observed all ape species for the same number of hours, and we report preliminary species comparisons, our data set cannot be used to draw conclusions about rates of teasing across species.

### (c) Identifying teasing events

**Figure 1.**
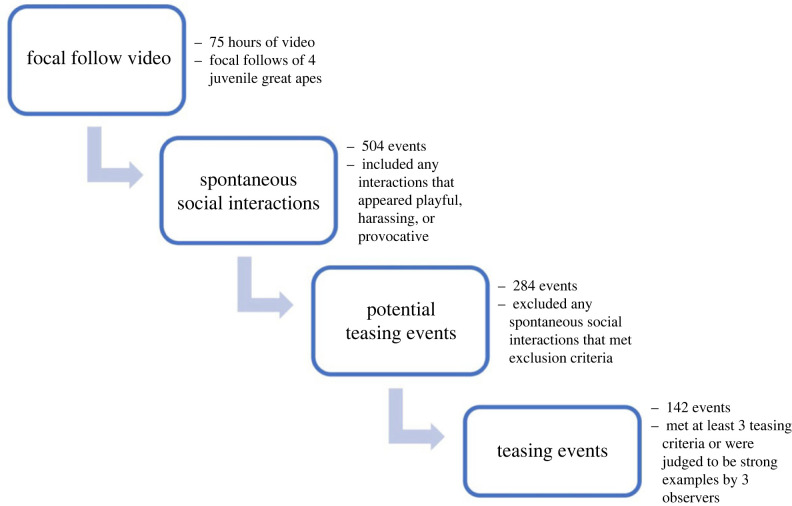
Diagram of the process for identifying and selecting playful teasing events for analysis.

We analysed 75 h of video (evenly split across species; mean = 18.8 ± 1.7 h) and extracted clips containing any spontaneous social interactions that appeared playful, harassing, or otherwise *provocative.* We excluded interactions that appeared explicitly aggressive without any provocative elements. See electronic supplementary material, section C, table S2 for detailed criteria. This process yielded 504 interactions that we analysed further to select potential teasing events. We removed interactions that (i) were mechanically effective, (ii) had an immediate adaptive benefit, (iii) were not directed towards another individual, (iv) were aimed at acquiring food, (v) occurred in rough-and-tumble play, (vi) were multi-party interactions, (vii) had a game-like structure, (viii) were embedded in other teasing events (teasing by the target in response to being teased), (ix) involved social behaviour that was clearly neither teasing nor play (e.g. grooming), and (x) had insufficient video quality. In contrast to Adang [20–22], we did not exclude behaviours directed towards juveniles or between adults (Adang was only interested in juvenile quasi-aggressive behaviour directed towards adults). See electronic supplementary material, section D, table S3 for details on these criteria used to exclude videos from the final set. This process yielded 284 potential teasing events, which we (IBL) coded for the presence of predefined teasing behaviours (see below for definition). Of the 284 potential teasing events, 129 met 3 or more of our behavioural criteria for teasing (described in the next section T1–5) and were considered ‘strong’ examples of teasing. We also included 13 teasing events that only met two of our teasing criteria but were judged to be admissible strong examples of teasing by all three coders (IBL, SW and EAC). These 142 strong examples of teasing (hereafter, ‘teasing events’) had clear markers of intentionality and were the target of our analysis (figure 1).

### (d) Behavioural coding

We developed a set of behavioural criteria to measure the behaviour of both teaser and target during a potential teasing event. We also recorded whether the events met several criteria of play in nonhuman animals (table 1; e.g. [35,36]; note that we are not making claims about the intentionality of the play criteria).

**Table 1.** Overview of the teasing (T1–T5) and play criteria (P1–P5). A criterion was considered met if any of the sub-criteria were met.

	criteria	sub-criteria	description/definition
T1	one-sidedness	a	Initial teasing/action sequence is one-sided, action coming mostly from teaser.
		b	Interaction was one-sided, unreciprocated or minimally reciprocated for the entire teasing event.
T2	surprise initiation		Element of surprise at the beginning (target is turned away, approach from behind).
T3	audience checking		Teaser seems to look for a response/looks towards target's face.
T4	provocative behaviour	a	Dominance display and species-typical displays (e.g. chest beat, drumming).^1^
		b	Waving/swinging body part or object in target's field of vision; action is slower, more relaxed, and less aggressive than dominance display.
		c	Other (e.g. doing something very slowly, stealing an object, staring).
T5	persistence/goal-directedness	a	Repetition of a behaviour.
		b	Elaboration (varying behaviour following no/minimal response) and/or escalation (behaviour increases in speed or size following no response).
P1	mutual enjoyment and play signals	a	Clear evidence of mutual enjoyment (both appear in a playful, relaxed state, no obvious tension).
		b	Actor produces one or more of the following play signals: playface, head-butt, chest beat (gorillas), laughter, somersault, place object on head. We also coded arm raise (without or with object) as a play signal if accompanied by other markers of play.
		c	Recipient produces one or more play signals.
P2	role reversal		Target directs a teasing action towards the teaser.
P3	rough and tumble play		Mock-biting, wrestling, chasing, tickling.
P4	voluntary initiation		Both individuals appear to engage voluntarily.
P5	relaxed context		Interaction starts as both individuals are relaxed (resting, feeding, foraging, relaxed locomotion, or grooming).

Almost all the teasing events we observed contained one or more of 18 actions we termed ‘teasing behaviours’. These were provocative behaviours directed from one animal (the teaser) toward the other (the target). We described them as provocative because they were difficult to ignore. They were typically tactile actions that moved the target's body or repetitive actions that took up a large fraction of the target's visual field. The 18 teasing behaviours were body slamming, hiding, hindering activity, hindering movement, hitting (both play-hitting and agonistic-hitting), hitting with object, offer and withdrawal of a body part, offer and withdrawal of an object, poking, poking with an object, pulling on a body part, pulling on hair, stealing (without immediate adaptive benefit, see electronic supplementary material, section D, table S4), swinging close, swinging an object, tickling, tug of war, and violating personal space (e.g. quickly leaning in close to another's face). Teasing events could include multiple teasing behaviours, and most teasing behaviours were mutually exclusive at any given time, except for hinder movement/activity and violate personal space, which could be done via one of the other behaviours or on their own. See electronic supplementary material, table S4 for an ethogram with definitions of these behaviours.

We also coded whether the teaser or target approached the other and the behavioural context immediately preceding the teasing event (see [20–22]). If the target's behaviour was disrupted, we coded their behaviour before and after the disruption (electronic supplementary material, table S5). We coded the target's responses to teasing (see [20–22]) as either affiliation, play, ignoring, moving away, mild aversion, or aggression (electronic supplementary material, table S6). Following the ape gesture coding conventions (e.g. [31]), we also indicated the target's first response if multiple responses occurred.

### (e) Statistical analysis

We conducted interobserver reliability on whether to include interactions as teasing events and found substantial agreement (Cohen's kappa ≥ 0.774; *z* = 5.2; *p* < 0.001 [37,38]). To establish reliability for our detailed behavioural coding of teasing, 39% of the 142 teasing events were re-coded by a second observer (SW). Interobserver reliability ranged from perfect to moderate agreement across the 51 coded variables (see SI, section E).

We used generalized linear mixed models (GLMMs) [39] to analyse the relationships between teasing criteria, characteristics of the social interactions, and identity of the teaser and target.

To estimate the extent to which the number of teasing criteria affected the probability of *mutual enjoyment* we fitted a GLMM with binomial error distribution and logit link function. In addition to the fixed effect of number of teasing criteria we included random intercept effects for teaser, target, teaser-target dyad, and species. We included ID as a random intercept effect because the data included several actors and targets as part of multiple dyads. The data used to fit this model comprised 118 strong teasing events involving 20 targets and 16 actors forming 37 dyads across 4 species (we excluded events in which the actor or recipient were not identifiable).

To estimate the probability that the teaser or the target was in a *relaxed state* before the teasing event happened, we fitted two GLMMs with binomial error distribution and logit link function, one that predicted whether the teaser was relaxed (teaser relaxed model) and one that predicted whether the target was relaxed (target relaxed model). Both models comprised only the intercept in the fixed effects part and species as a random intercepts effect. Furthermore, we included the identity of the teaser (teaser relaxed model) or the target (target relaxed model) as additional random intercepts effects. We assessed the significance of the fixed effects intercept. If the teaser or target was significantly more likely to be relaxed than not relaxed, the intercept in the respective model would be significantly greater than zero (relaxed = 1, not relaxed = 0). The teaser relaxed model comprised 109 observations involving 16 teasers of 4 species, and the target relaxed model comprised a total of 111 events involving 20 targets of 4 species. Sample sizes differ because in some cases the identity of the teaser or the target was not known.

To assess whether juveniles or adults were more likely to initiate teasing events, we evaluated events in which the actor and the recipient were not of the same age class. The GLMM failed to produce reasonable results with regard to the random effects because the actor always initiated the teasing events. So, instead, we calculated the proportion of teasing events each individual initiated out of all those they were involved in. We then compared this proportion between juveniles and adults using an exact Mann–Whitney *U*-test [40,41].

Finally, we estimated the probabilities of different *first responses* (affiliation and play, ignoring, moving away, mildly aversive) using a multinomial GLMM. We excluded aggression as it occurred too rarely. We included random intercepts effects for teaser, target, teaser-target dyad, and species (*n* = 115 events, 16 teasers, 20 targets in 37 dyads of 4 species).

We fit all models in R (version 4.1.2; [42]). Binomial models were fit using the function glmer of the package lme4 (version 1.1–28; [43]), the multinomial models were implemented in a Bayesian framework using the function brm of the package brms (version 2.16.3) [44–46], and the Mann–Whitney *U*-tests were calculated using the function Wilcox exact of the package exact rank tests (version 0.8–34) [47]. For binomial GLMMs, we estimated model stability by dropping individual teasers, targets, dyads and species one at a time, fitting the respective model to all subsets, and comparing the range of estimates obtained with those of the full model. For these models, we obtained confidence intervals of model estimates by means of a parametric bootstrap (*n* = 1000 bootstraps; function bootMer of the package lme4).

## 3. Results

We started with the same number of hours of video for each ape species, but the 142 strong examples of teasing in our final dataset were asymmetrically distributed across species (*n*_chimpanzee_ = 84, *n*_orangutan_ = 31, *n*_bonobo_ = 20, *n*_gorilla_ = 7). We mainly present results using a pooled dataset, acknowledging that the majority of teasing events come from chimpanzees. The chimpanzee group was the largest group we observed (*n* = 20), which may have increased the likelihood of interactions due to a greater number of potential social partners; see ‘Limitations of the study’ below).^2^ Due to our sampling method and sample size, we make no claims about species differences. However, we do report the teasing behaviours we observed across species and age classes for the purpose of generating hypotheses for future studies.

### (a) Provocative elements

We identified 18 different teasing behaviours (table 2; electronic supplementary material, section D, table S4 for definitions of each behaviour). On average, teasers performed more than one teasing behaviour during a teasing event (mean = 1.36, SD = 0.56). Many of these behaviours appeared to be designed to provoke a response, or at least to attract the target's attention. For example, it was common for teasers to repeatedly wave or swing a body part or object in the middle of the target's field of vision, hit or poke them, closely stare at their face, disrupt their movements, or steal objects for no obvious functional purpose (table 2). As a proposed criterion for teasing (table 1, T4), we loosely grouped these seemingly provocative behaviours into (i) whole body dominance displays (T4a, 6% of events), (ii) waving objects or self (T4b, 22% of events), or (iii) other attention-getting behaviours like leaning into the target's face and staring (T4c, 16% of events).

**Table 2.** Number of teasing behaviours shown by each species. Behaviours are sorted by overall frequency and the five most frequent behaviours for each species are underlined. All orangutan and gorilla teasing behaviours were produced by a single individual (focal juvenile). Chimpanzee and bonobo teasing behaviours were produced by multiple individuals (chimpanzees: 2 juveniles, 7 adult females, 2 adult males; bonobos: 1 juvenile, 2 adult females). The data are pooled to highlight the distribution of teasing events across the dataset. Behaviours by individual are presented in electronic supplementary material, tables S12 and S13.

teasing behaviours	bonobo	chimpanzee	gorilla	orangutan	total
poking	5	46	5	19	75
hitting	5	31	2	15	53
hindering movement	8	17	3	11	39
body slam	8	15	2	5	30
pulling on body	2	15	1	9	27
swinging	6	6	1	10	23
violating personal space	5	3	2	5	15
tickling	1	12	0	0	13
pulling on hair	0	2	1	10	13
stealing	0	11	0	2	13
swinging of object	2	4	0	6	12
hindering activity	2	9	0	0	11
hitting with object	1	6	0	3	10
tug of war	0	6	0	2	8
poking with object	0	3	0	0	3
offer + withdrawal object	0	1	0	2	3
offer + withdrawal body	0	2	0	0	2
hiding under an object	0	0	0	1	1

**Figure 2.**
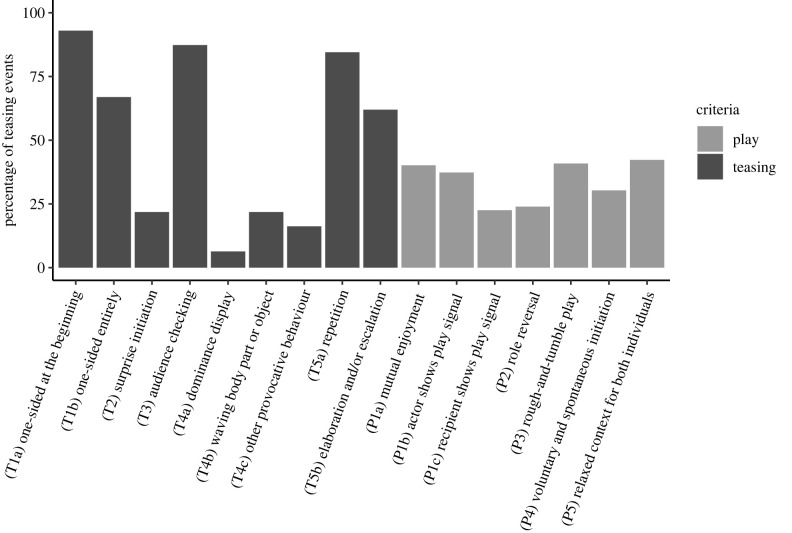
Bars represent the frequency of teasing criteria (T1–T5; dark grey) and play criteria (P1–P5; light grey) observed across teasing events (*n* = 142) (see table 1 for criteria definitions). Teasing events were most typically characterized by one-sidedness at the start (T1a; 92%), one-sidedness throughout (T1b; 66%), audience checking (T3; 87%), repetition (T5a; 84%), and elaboration and escalation (T5b, 62%).

Overall, teasing events were primarily characterized by one-sidedness (T1), looking for a response (T3), repetition (T5a), elaboration and escalation (T5b; figure 2). Examples are shown in the electronic supplementary material, movie S1. Teasing events that fulfilled 4–5 teasing criteria lasted longer than teasing events that met only 2–3 criteria (Mdn_4–5 criteria_ = 40 s, IQR = 69–22 s; Mdn_2–3 criteria_ = 26 s; IQR = 55–11 s). Ninety-two per cent of teasing events were one-sided in the beginning of the interaction, and most teasing events (66%) continued to be one-sided or only minimally reciprocated during the entire event. In 87% of events, the teaser looked towards the target's face during or immediately after a teasing behaviour or sequence. If the target showed minimal/no response, the teaser typically repeated the action (84%) or persisted through elaboration and/or escalation (62%). Twenty-two per cent of events contained surprise initiation, in which the teaser approached from behind or produced the initial teasing behaviour while the target was facing away (T2).

### (b) Intentionality

To assess intentionality, we looked for evidence of *directedness* and *persistence* in the teaser's behaviour. All teasing events in our final dataset were directed towards a specific individual. In most events (62%), the teaser approached the target within 30 s of the first teasing behaviour. In 26% of events, the teaser and target were already in proximity before the teasing began, and in only 7% of events the target approached the teaser (in 5% the approach was unclear). Teasers showed high levels of persistence by repeating the same behaviours (T5a; repetition: 84%) and/or by increasing the intensity of the behaviour (T5b; elaboration and/or escalation: 62%; figure 2). The teaser looked towards the target's face in the majority of events (87%), seemingly expecting a response (figure 2, T3).

### (c) Playful elements

Teasing events displayed several play markers (figure 2, P1–P5). The vast majority of teasing events in our sample (87%) met at least one play criterion (table 1, P1–P5). Play-specific signals were produced in the minority of instances: by the teaser in 37% of events (P1a) and by the target in 22% (P1b). Forty percent of events were characterized by mutual enjoyment, in which both apes appeared to be in either a playful or relaxed state (P1c). We did not find an obvious effect of the number of teasing criteria on the probability of mutual enjoyment (*χ*^2^ = 0.083, df = 1, *p* = 0.733; electronic supplementary material, section F, table S9). In roughly one quarter of events (24%), we observed role reversal (P2), in which the target later directed a teasing behaviour towards the teaser. Forty-one per cent of events contained typical rough and tumble play (P3), such as mock-biting, wrestling, chasing and/or tickling. Thirty per cent were voluntarily and spontaneously initiated by both individuals (P4). In 42%, both animals were in a relaxed state before the start of the interaction (P5).

### (d) How teasing differs from play

**Figure 3.**
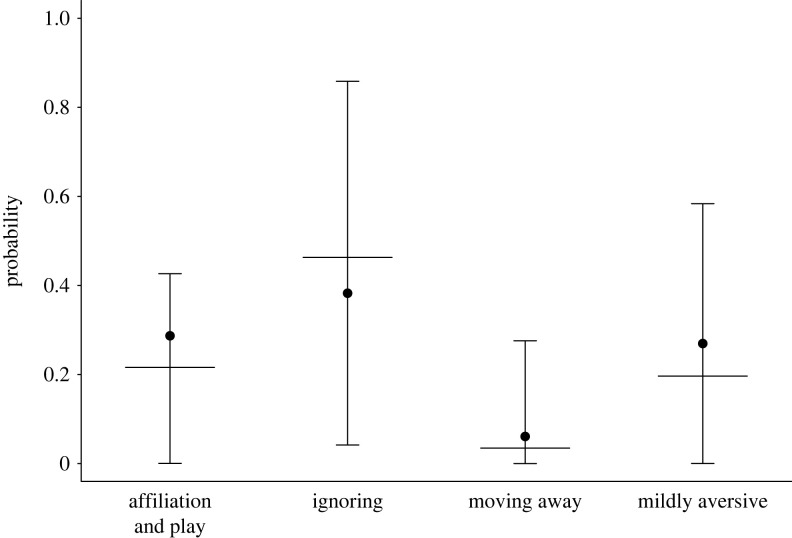
Probability of different first responses to teasing. Dots depict means and error bars depict the estimated means and their 95% confidence intervals from the multinomial GLMM.

While the teasing events we observed contained many elements of play, they differed from play in important ways: primarily in their asymmetry, incorporation of surprise, and infrequent use of play-specific signals like the primate playface. They also rarely displayed movement final ‘holds’ characteristic of play-initiating gestures in great apes [31–33]. Pure play is typically highly reciprocal, whereas most teasing events in our sample were one-sided from the start (92%) and often continued to be asymmetric during the entire event (66%). Teasing events were also unlikely to result in play; only 26% of teasing events resulted in play or other types of affiliative actions, like grooming (figure 3).

We must also consider the possibility that playful teasing events are unsuccessful play requests. Failed play requests would also appear as one-sided interactions characterized by markers of intentionality and accompanied by play signals. However, we believe that it is possible to differentiate between failed play requests and playful teasing by the lack of species-typical play-initiating gestures and the absence of movement-final holds. All species of great apes have species-typical gestures that are used to initiate play (e.g. hand raise, playface, head-butt, chest drumming, etc. [31–33,48]). These are robust gestures found across different populations. Play-initiating gestures are particularly common in captivity, where they can represent more than 50% of the gestures in a species's reported repertoire [31]. Play signals like the playface expression were observed in 53 out of the 142 teasing events, but many play-specific gestures were entirely absent from our data. It was rare for the teaser to produce a play signal (for list see table 1) when initiating a teasing event (10% of all events), and in these cases, only one event showed the movement-final ‘hold’ characteristic of many gestures.

Teasing often elicits ambiguous behaviour in the recipient. We coded both the first response to teasing and the change in response during a teasing event. Targets often displayed several responses because teasers typically produced several teasing behaviours per event. For example, an adult might respond to repeated hitting by first ignoring, then ducking, ignoring again, and finally moving away. The most common overall responses to teasing behaviours were neutral to mildly aversive: ignoring the teaser (27%), mildly aversive behaviour (24%) or moving away (17%). Recipients occasionally responded with affiliative behaviour (13%) or play (14%) at some point during teasing events. Aggressive responses were seen in only 5% of teasing events, and the first response was aggressive in only 4%. The target's first response to teasing was typically neutral. Affiliative and neutral first responses were more common than aggressive ones or moving away (figure 3; note that we excluded aggression as a first response in the model as it occurred too rarely). The target's *first response* was ignoring in 41% of events, play and affiliation in 26%, mildly aversive behaviour in 24%, moving away in 5%, and aggression in 4%.

### (e) Relaxed behavioural contexts

Teasing occurred most often in relaxed behavioural contexts. We coded the behavioural context of both teaser and target in the five seconds before approach and/or first teasing behaviour (for details see electronic supplementary material, section D, table S5). Before the teasing began, the target was more likely to be engaged in relaxed behaviours than in highly arousing behaviours such as play, teasing, mating, object manipulation, fast locomotion, aggression or agitation (test of the average intercept estimate in the target relaxed model was significantly greater than 0: Z = 3.517, *p* < 0.001; see electronic supplementary material, section F, table S10). The target was in a relaxed behavioural context before 73% of teasing events, including resting (44%), relaxed locomotion (22%), feeding and foraging (5%), and affiliative behaviour (2%). In 6% of events, the behaviour could not be coded, usually because the target was not fully visible. Prior to teasing, the teaser was also more likely to be engaged in relaxed behaviours than arousing behaviours (test of the average intercept estimate in the teaser relaxed model: Z = 2.702, *p* = 0.007; electronic supplementary material, section F, table S10). We observed the teaser in a relaxed context prior to 61% of the events, including relaxed locomotion (27%), resting (26%), feeding and foraging (6%) and affiliative behaviour (2%). In 7% of events the context could not be coded.

We also coded whether the teasing event disrupted an activity that the target had been engaged in before the teasing began or otherwise significantly altered the target's behavioural state. In 58% of teasing events, the teaser disrupted the target's relaxing/resting. In 23%, the teaser stopped or initiated the target's movement by hindering their locomotion, deviating their path, or initiating their locomotion. In 12%, the teaser disrupted high intensity or high focus behaviours, such as tool-use, object manipulation, solitary or social play. In only 2% of events, the teaser did not interrupt or cause any visible behavioural state change in the target. In 6% of events, we were unable to code behavioural change due to poor visibility.

### (f) Similarity of teasing behaviours across species

Although the four great apes have very different socioecologies, they tease in largely similar ways. The majority of teasing behaviours were seen in at least three species: chimpanzees (17 out of 18 teasing behaviours), orangutans (14 out of 18) and bonobos (11 out of 18; table 2). Gorillas used only 8 of the 18 teasing behaviours. It is unsurprising that chimpanzees displayed almost all of the teasing behaviours we documented, since chimpanzees produced 59% of our dataset. Three teasing behaviours (poking, hitting, hindering movement) were within the top five most frequent behaviours for all species (table 2). Across species, the most common teasing behaviours were poking (21%), hitting (15%), hindering a conspecific's movement (11%), body slamming (9%; either gentle or strong hitting with the whole body) and pulling on a conspecific's body part (8%). Together, these five teasing behaviours accounted for 64% of all teasing behaviours.

### (g) Potential species differences

**Figure 4.**
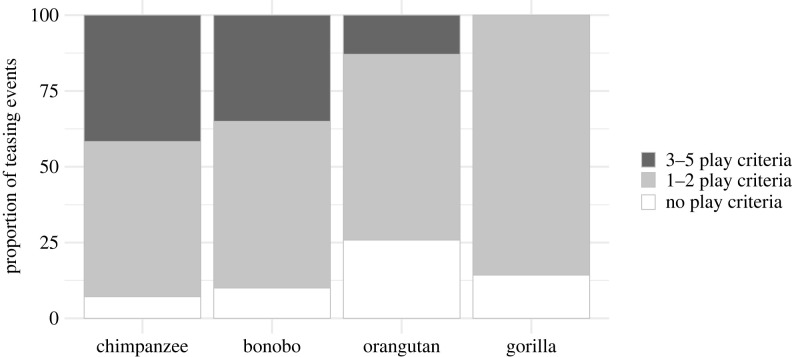
Proportion of teasing events that met 3–5 (dark grey), 1–2 (light grey), and none (white) of the five play criteria for each great ape species (*n*_chimpanzees_ = 84, *n*_bonobo_ = 20, *n*_orangutan_ = 31, *n*_gorilla_ = 7).

Across species, around half of all teasing events (49%) met at least one play criterion, and 37% met three or more (figure 4, table 1 for play criteria). A large proportion of teasing events in chimpanzees (48.8%) and bonobos (40%) fulfilled three or more of the five play criteria, whereas only 13% of orangutan teasing events and no gorilla teasing events fulfilled more than two. This suggests that teasing could involve different levels of enjoyment or playfulness across the different species, or at minimum that non-aggression is signalled in different ways across species.

### (h) Potential age-related differences

Our initial video corpus focused on juveniles because it was recorded using focal follow sampling of juveniles. However, one of the two individuals involved in teasing events was typically an adult (94% of all events). This means that in at least 134 events (the number of events involving both a juvenile and an adult), either the adult or the juvenile could have initiated teasing. In these 134 events, juveniles initiated teasing more often (exact Mann–Whitney *U*-test, two-tailed: *W* = 3.5, *n*_adult_ = 11, *n*_juvenile_ = 5, *p* < 0.001; adults were the initiators in 38, juveniles in 96). Though the most common social partner for juvenile apes is their mother [49], mothers were not common targets of teasing (for details on who was teasing whom see electronic supplementary material, section G, table S11 or discussion). The orangutan juvenile ‘Aisha’ and the bonobo juvenile ‘Belle’ never teased their mothers. The chimpanzee juvenile ‘Azibo’ teased his mother only 14% of the time, and the juvenile ‘Ohini’ teased her mother only 8% of the time. The exception was the gorilla juvenile ‘Denny’, who directed his teasing behaviours towards his mother or his father most of the time (71% and 14% respectively); however this was the smallest group, with only four individuals.

When an adult was observed initiating a teasing event (*n* = 40; including those between 2 adults), it was overwhelmingly a chimpanzee (93%, *n* = 37) and directed toward a juvenile (95%, *n* = 38). Adult teasers were more often female (*n* = 19) than male (*n* = 10; one unknown sex), but this may have been an artefact of group composition and sampling. Our sample contained only two teasing events between adults: both occurred in chimpanzees and involved male teasers. Although our sample of adults is influenced by the focal sampling method and number of adults in each group, it is still notable how few adult-adult teasing events were observed, particularly since many videos included more than two adults visible for extended periods of time.

We found that the five most frequent teasing behaviours differed somewhat between adults and juveniles. Juveniles teased primarily by poking (20%), hitting (18%), hindering movement (12%), body-slamming (11%), and swinging or waving a body part (9%). Adults teased mainly by poking (25%), tickling (14%), pulling on a body part (11%), hindering movement (9%), and stealing (9%). In both juveniles and adults, the top five actions accounted for around 70% of all the observed teasing behaviours (for details of all actions by age class see electronic supplementary material, section H, tables S12 and S13). Overall, adults seemed to tease using gentler actions than juveniles. Adult chimpanzees were more likely to poke than hit a juvenile (*n*_poke_ = 23 versus *n*_hit_ = 7), whereas juveniles were equally likely to poke or hit an adult (*n*_poke_ = 23 versus *n*_hit_ = 23). Adult bonobos never hit and only once poked the juvenile.

Juveniles were more likely than adults to use explicit play signals during teasing. In teasing events involving juvenile teasers, the teaser tended to show more play signals (for description table 1) than the target (Wilcoxon test, V = 15, *n* = 5, *p* = 0.0625; chimpanzee: 69% versus 17%, *n*_Azibo_ = 35; 58% versus 17%, *n*_Ohini_ = 12, bonobo: 65% versus 12%, *n* = 17, orangutan: 19% versus 3%, *n* = 31, gorilla: 14% versus 0%, *n* = 7). This pattern was observed in adult bonobos as well (67% versus 33%; *n* = 3), but not in adult chimpanzees, in which this trend was reversed. In events involving an adult chimpanzee teaser, the target was more likely than the teaser to use play signals (56% target versus 5% teaser; *n* = 37).

## 4. Discussion

Our results support the idea that teasing in great apes is a *provocative*, *intentional* and often *playful* behaviour. It is typically asymmetric and can take different forms with varying proportions of playful and aggressive features. Similar to teasing in human infants, playful teasing in great apes contains response looking, repetition and elaboration, and mostly takes place in *relaxed* contexts. We found few *species differences,* but several age-related differences: the four species teased in largely similar ways, but juveniles appeared to tease more than adults and often employed different behaviours. It seems likely that there is an interaction between age and species, as we observed different patterns of play signal use in adult and juvenile chimpanzees and bonobos. However, caution is warranted in drawing any species-wide trends due to our sampling limitations (see ‘Limitations of the study’).

### (a) Provocative

In our observations, teasers led the interaction. Most teasing events started one-sided (92%) and most were asymmetric throughout the entire event (66%). Twenty-two per cent of the teasing events contained an element of surprise, like an approach from behind followed by a startled response from the target. Surprise is also common in human infant teasing [2]. The teaser looked towards the target's face in most events (87%), presumably to monitor their response. This echoes findings in human infants as young as 7 months old [50]. Since teasing walks a fine line between aggression and play, facial cues might be important to predict the target's responses, allowing the teaser to adjust accordingly [10]. Given that targets reacted only very rarely with aggression, it is likely that the teasers used appropriate signals or otherwise adapted their behaviour to avoid misinterpretation and escalation into serious aggression.

While we cannot be certain that all of the behaviours we observed were driven by a motivation to provoke a response, we argue that teasing is the best characterization of the interactions we observed. We only included events that seemed to be directed towards eliciting a social response, and we explicitly excluded events that included requests for food or objects that were then immediately used by the teaser. For example, we only included object stealing events in which the teaser immediately lost interest in the object and that also met three or more of our teasing criteria (see exclusion criteria in electronic supplementary material, section D, table S3).

### (b) Intentional

The teasing events we observed met widely-accepted criteria of intentionality for ape gestures [31,32,34]. Teasing was always directed towards a specific target. The teaser typically approached the target shortly before the first teasing behaviour. Teasers showed persistence when the targets ignored them (or only minimally reacted), typically by repeating their behaviour (in 84% of events), or elaborating by increasing intensity or switching behaviour (in 62% of events). The wide range of actions used to tease others (18 behaviours in total) suggests that playful teasing in great apes can take many different forms. This is similar to the flexibility seen more broadly in play, which tends to co-opt behaviours from other contexts and use them in modified forms [35].

### (c) Playful

Most of the teasing events in our sample met at least one (but usually several) play criteria (play signals, role reversal, rough and tumble play, voluntary initiation, relaxed context; figure 4). Signals like the playface may reduce uncertainty in recipients by signalling playful intent, thus lowering the risk of misinterpretation of play as aggression and facilitating the coordination of playful behaviours [27–29]. It is likely that play signals function similarly within playful teasing since teasing behaviours can be highly ambiguous. Note that while we argue playful teasing is an intentional type of behaviour, facial signals of play are not necessarily intentional (they could be reflections of internal affective states).

While teasing events in our sample were *playful*, they were distinct from typical play in that they showed low rates of play signals, were highly asymmetrical, and overwhelmingly occurred between individuals with very different body size or social status.

In our sample, play signals were produced less than half the time (by the teaser in 37% and the target in 22%) and only rarely at the start of a teasing event (the teaser used a play signal only 10% of the time when initiating teasing). Critically, almost all behaviours accompanied by play signals lacked the movement-final ‘hold’ characteristic of intentional play-initiating gestures. The majority of playful teasing events in each species met only one or two of our five play criteria [35,36] (figure 4). Importantly, species-typical gestures used to initiate play [31–33,48] were largely absent from playful teasing events, suggesting that playful teasing is not just failed attempts to initiate play.

In studies of social play, initiating play is usually positively correlated with receiving play (e.g. western lowland gorillas [51]; dogs [52–54]). This suggests that play is a highly reciprocal behaviour, whereas most teasing events in our sample were largely one-sided. Targets of teasing responded with play or other affiliation only a quarter of the time. In most cases, the target either ignored the teaser or reacted with mild aversion or moving away. Given that in 74% of teasing events, the target showed neutral or negatively-valanced behaviour towards the teaser as a first response, playful teasing seems distinct from attempts to initiate play.

When a target responded to an initial teasing behaviour by mildly rebuffing the teaser, apes were still likely to persist by repeating or elaborating. This persistence in the face of negative feedback differs somewhat from human infants, who rarely repeat teasing behaviours that receive negative responses [8], though it is unclear if the mildly-aversive responses we observed would be considered negative in humans. Of course, human teasers sometimes persist when faced with negative responses, as in the case of taunting and bullying.

Finally, according to the motor-training hypothesis of play, juveniles are expected to play primarily with individuals that match them in body size and social skills (e.g. [55,56]). This is consistent with many reports of animal play (e.g. [57,58]). While group composition did not allow us to systematically test this in all species, qualitatively, we saw the opposite pattern in playful teasing. Apes were more likely to tease individuals who differed from them in size and social skills (i.e. teasing events were most likely to involve one juvenile and one adult).

Taken together, these features support our argument that playful teasing is not merely an unsuccessful attempt to initiate play, but rather is a distinct type of behaviour.

### (d) Relaxed

Teasing in our sample typically occurred in relaxed contexts (73%), similar to studies of human infants [2]. This suggests that primate teasing is not primarily aggressive (as argued by previous work [20–26]), but instead may occur during moments of neutrality or even boredom. In our sample, teasing almost always began with the target engaged in a relaxed behaviour (e.g. rest), and often led to a change in behaviour. Occasionally, the teaser changed the target's locomotion, but teasing rarely disrupted high intensity or highly focused activities, like tool use or object manipulation.

Roughly 40% of teasing events were judged to be mutually enjoyable for the majority of the interaction (i.e. both apes were in a playful and relaxed state). Hence, a function of playful teasing may be to strengthen social bonds by creating a shared positive experience, as has been suggested for teasing in human infants (e.g. [2,59]). However, only a quarter of teasing events ended with overtly prosocial responses from the target, like play or affiliation.

### (e) Species continuity

Even though our data do not allow us to make robust claims about species differences (see limitations of the study) and the majority of playful teasing events come from chimpanzees, we nevertheless observed that the four great ape species in our sample teased in similar ways. All species primarily teased by poking, hitting or hindering movement. These actions have been described in previous studies focused on agonistic forms of teasing [20–26] or in the context of play (e.g. [60]). We observed differences in the ways species incorporated elements of play into teasing events. In our sample, chimpanzee and bonobo teasing displayed more of our five play criteria than orangutan or gorilla teasing. Some differences among species could potentially be explained by differences in play style or bodily affordances: for example, pulling hair was common in orangutans, who have long hair, but was not commonly observed in the other species.

### (f) Age and sex

Juveniles were more likely to playfully tease others than adults. This is consistent with previously reported patterns in agonistic teasing [20–26,61]. This juvenile skew may also be explained by a developmental decline in play behaviour, which decreases with age as foraging time and sexual behaviour increase [62]. We also saw species differences in whom the individuals targeted (with the caveats of limited sample size and group composition). Juvenile primates are most likely to initiate interactions with their mothers [49], and juvenile-mother pairings are typical in ape social play [63]. In our study, however, most playful teasing events were not directed towards parents (see electronic supplementary material, section G, table S11). There may also be species differences that reflect socioecology. Playful teasing was mostly directed towards adult males in chimpanzees and to adult females in bonobos. This suggests that playful teasing has different functions than those proposed for agonistic teasing, which predicts teasing should be directed towards low-ranking adults. For example, the focal male juvenile chimpanzee was more likely to tease adult males than adult females (mother excluded). This is inconsistent with Nishida's rank improvement hypothesis [23], which predicts that young male chimpanzees should use teasing (harassment) towards adult females to improve their rank. Our chimpanzee data were not consistent with the rank-improvement hypothesis, but our bonobo data were. In bonobos, the juvenile was most likely to tease the lowest-ranking female, consistent with a rank-improvement or rank-enforcement function.

We observed some differences in the teasing behaviours used between adults and juveniles. In chimpanzees, adults were more likely to poke than hit juveniles, whereas juveniles were equally likely to hit or poke adults, suggesting that adults may self-handicap when playfully teasing juveniles. In studies of animal play, self-handicapping is often used when playing with same-aged or younger conspecifics (e.g. [64–67]). This strategy is believed to increase the likelihood of successful play in partners of mismatched power or ability [68]. In our study, adults may have poked rather than hit juveniles in order to increase the likelihood that their actions would be perceived playfully.

From an evolutionary perspective, the presence of playful teasing in all four great apes and its similarities to playful teasing and clowning/joking behaviour in human infants suggests that playful teasing and its cognitive prerequisites may have been present in our last common ancestor, at least 13 million years ago [69]. These findings have implications not only for primatologists and biological anthropologists, but for the study of emotion, humour and pretense more broadly. We hope that our study will inspire other researchers to study playful teasing and equip them with coding criteria to document playful teasing in other species in order to better understand the evolution of this multi-faceted behaviour.

### (g) Limitations of the study

As with most studies of primate behaviour, our study is limited by the composition and size of our sample. Because we used ad libitum sampling, this study cannot be used to make claims about relative rates of playful teasing or the relative likelihood of playful teasing across species. However, we found that all four great ape species use a wide range of behaviours to tease others. This suggests that playful teasing in great apes is not limited to a few species-typical actions, but instead can take many forms, perhaps with varying levels of playful and aggressive elements. To fully capture and understand this variation, it would be important to study playful teasing behaviour in several groups of each great ape species (ideally in both zoo-housed and wild apes). This would allow researchers to ‘tease’ apart species and group differences. In our study, both Pan species produced more playful teasing events than orangutans or gorillas did (though it is important to note that all orangutan and gorilla teasing events were produced by a single individual). As we only analysed playful teasing behaviour in one captive group of each species, we cannot be confident that these are reliable species differences. Factors like group composition, group size, sex and age ratios, and individual differences may play a role in the presence of playful elements in teasing events and may have shaped the form, distribution, and frequency of the behaviours we report.

## 5. Conclusion

Playful teasing in great apes is an intentionally provocative, asymmetric behaviour with varying proportions of playful and aggressive elements. Similar to teasing in human children, playful teasing in apes involves one-sided provocation, response waiting, elaboration, repetition and elements of surprise. Playful teasing mainly occurred in relaxed contexts and resulted in neutral outcomes. We found that species teased in similar ways, and that juveniles teased more than adults. Playful teasing in great apes is most likely a homologous trait, inherited from our last common ancestor, which possessed cognitive precursors for joking and humour.

## Data Availability

The data are provided in electronic supplementary material [70].

## Endnotes

1 We originally included another subcategory of provocative behaviour: 'Initiating contact in a provocative way (e.g. violating personal space, blocking someone's way, hitting, poking, pulling fur, approaching very close).' However, we were unable to reach sufficient interobserver reliability. Nonetheless, we were able to reach reliability on the actions (e.g. poking, hitting) comprising this subcategory. Future researchers might benefit from including similar behaviours.

2 The chimpanzee group had more individuals and thus more potential interactions, but when we consider teasing events which involve only the focal individual, we still find that 53.6% of our dataset comes from the chimpanzee, with 24.8% from the orangutan, 16% from the bonobo, and 5.6% from the gorilla.
